# Methylation of H2AR29 is a novel repressive PRMT6 target

**DOI:** 10.1186/1756-8935-4-11

**Published:** 2011-07-20

**Authors:** Tanja Waldmann, Annalisa Izzo, Kinga Kamieniarz, Florian Richter, Christine Vogler, Bettina Sarg, Herbert Lindner, Nicolas L Young, Gerhard Mittler, Benjamin A Garcia, Robert Schneider

**Affiliations:** 1 MPI for Immunobiology and Epigenetics, Stübeweg 51, 79108 Freiburg, Germany; 2 Innsbruck Medical University, Division of Clinical Biochemistry, Biocenter, Fritz-Pregl-Str. 3, 6020 Innsbruck, Austria; 3 Princeton University, Department of Molecular Biology, 415 Shultz Laboratory, Princeton, NJ 08540, USA; 4 University of Konstanz, Doerenkamp-Zbinden chair, Universitätsstr. 10, 78457 Konstanz, Germany

## Abstract

**Background:**

Covalent histone modifications are central to all DNA-dependent processes. Modifications of histones H3 and H4 are becoming well characterised, but knowledge of how H2A modifications regulate chromatin dynamics and gene expression is still very limited.

**Results:**

To understand the function of H2A modifications, we performed a systematic analysis of the histone H2A methylation status. We identified and functionally characterised two new methylation sites in H2A: R11 (H2AR11) and R29 (H2AR29). Using an unbiased biochemical approach in combination with candidate assays we showed that protein arginine methyltransferase (PRMT) 1 and PRMT6 are unique in their ability to catalyse these modifications. Importantly we found that H2AR29me2 is specifically enriched at genes repressed by PRMT6, implicating H2AR29me2 in transcriptional repression.

**Conclusions:**

Our data establishes R11 and R29 as new arginine methylation sites in H2A. We identified the specific modifying enzymes involved, and uncovered a novel functional role of H2AR29me2 in gene silencing *in vivo*. Thus this work reveals novel insights into the function of H2A methylation and in the mechanisms of PRMT6-mediated transcriptional repression.

## Background

Post-translational modifications of histones play an important role in the regulation of all nuclear processes occurring on chromatin. Depending on the type of modification and/or the residue modified, they can be involved in gene activation or silencing. In particular the methylation of histones, has been extensively studied, and has been shown to regulate both processes [1]. Histones can be methylated on lysine residues by lysine methyl transferases (KMTs) and on arginine residues by protein arginine methyl transferases (PRMTs). Of the four core histones (H3, H2B, H2A and H4), methylation of the N-terminal tails of H3 and H4 has been intensively studied, whereas very little is known about modifications of H2A and H2B.

Several potential methylation sites in H2A have been identified by mass spectrometry (MS) analysis, including the presence of at least two methyl groups in the first 17 amino acids of H2A [2]. However, the only methylation site of H2A that has been experimentally studied is methylation of arginine 3 (H2AR3) [3].

PRMTs are involved in a variety of cellular processes [4-6], and have recently been linked with carcinogenesis [7]. Multiple PRMTs have been described to date [8], all of which share a set of conserved sequence motifs (I, post-I, II and III) and a THW (threonine-histidine-tryptophan) loop, but differ in the composition of their protein domain and in their cellular localisation. All PRMTs can catalyse monomethylation of arginines (MMA), and are divided in two families according to their dimethylation activity: type I enzymes catalyse asymmetric dimethylation (aDMA), whereas type II enzymes perform symmetric dimethylation (sDMA), [4,5,9]. Of the type I enzymes, PRMT1 methylates R3 in H4 and H2A *in vitro *[3], and PRMT4 methylates H3 on R2, R17 and R26 [10]. However, the main PRMT able to methylate H3R2 *in vivo *is PRMT6 [11,12], and PRMT6 can also methylate H4 and H2A *in vitro *[11]. Of the type II PRMTs, PRMT5 methylates H4 and H2A on R3, and H3 on R8 [10]. PRMT7 catalyses monomethylation of histones *in vitro*, but the specific arginines targeted remain unidentified [13,14]. Recently, PRMT2 has been shown to methylate histones H4 and/or H3 [15,16]. For PRMT3, PRMT8 and PRMT9, no histone substrates have been described yet.

Modifications of histone H2A and the role of the H2A N-terminal tail itself in nucleosome biology are not fully understood. Because of this, we aimed to identify and characterise new methylation sites within H2A and their effectors. In this study, we identified H2AR11 and H2AR29 as novel arginine methylation sites, and showed that these modifications are set *in vitro *by the enzymes PRMT1 and PRMT6. To understand the function of H2AR29 methylation, we raised an H2AR29me2-specific antibody, and found that this marker is indeed *in vivo *catalysed by PRMT6. We found that H2AR29me2 is enriched on genes repressed by PRMT6, establishing H2AR29me2 as a novel repressive player involved in PRMT6 function.

## Results

The knowledge of H2A modifications is still very limited, and the characterisation of novel histone markers is fundamental to understand how post-translational modifications can regulate gene expression. H2AR3 is the only methylation site on this histone characterised to date [3,17], therefore we were interested in identifying novel H2A methylation sites, the modifying enzymes and their function.

### Endogenous PRMT1 can methylate H2A

**Figure 1:**
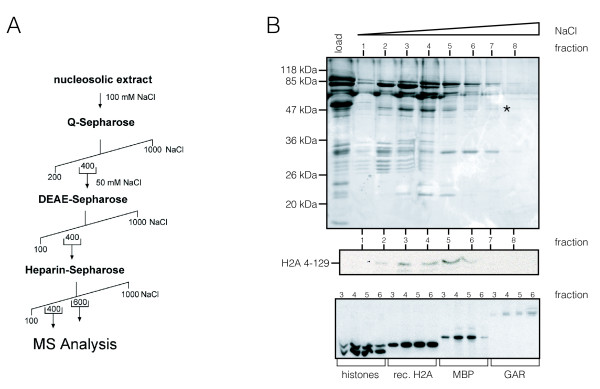
**H2A is methylated by protein arginine methyltransferase (PRMT)1 at novel sites**. **(A) **Biochemical purification scheme (for details see Additional file 5, supplementary materials and methods) of H2A-specific histone methyltransferase from HeLa nuclear extract. **(B) **(Upper panel) Silver stain of heparin-Sepharose fractions 1 to 8. PRMT1 was identified by mass spectrometry (asterisk) (see Additional file 1, Figure S 1). Autoradiography of histone methyltransferase assays of indicated heparin-Sepharose fractions using (middle panel) H2A 4-129 and (lower panel) endogenous acidic extracted histones, full-length recombinant H2A, myelin basic protein (MBP), and glutathione S-transferase (GST)-GAR (glycine- and arginine-rich) as substrates.

We followed an open and unbiased approach to identify histone methyltransferases that specifically methylate histone H2A. We established a biochemical fractionation protocol to purify these enzymes from nucleosolic HeLa cell extract (Figure 1A). To distinguish between H2AR3 methylation and methylation of other sites, we used N-terminally truncated H2A (H2A 4-129) as a substrate in our assays.

In the fractions eluted from the heparin-Sepharose column, we detected activities able to methylate recombinant full-length H2A, endogenous H2A in octamers, myelin basic protein (MBP), GAR (glycine- and arginine-rich) motifs (a generic substrate for PRMTs) (Figure 1B, lower panel), and most importantly, H2A 4-129 (Figure 1B, middle panel). MS analyses determined this activity as PRMT1 (Figure 1B, asterisk; see Additional file 1, Figure S1). Taken together, these results show that PRMTs, in particular PRMT1, can methylate H2A on sites other than R3.

### PRMT1 and 6 methylate H2A on R11 and R29 *in vitro*

**Figure 2:**
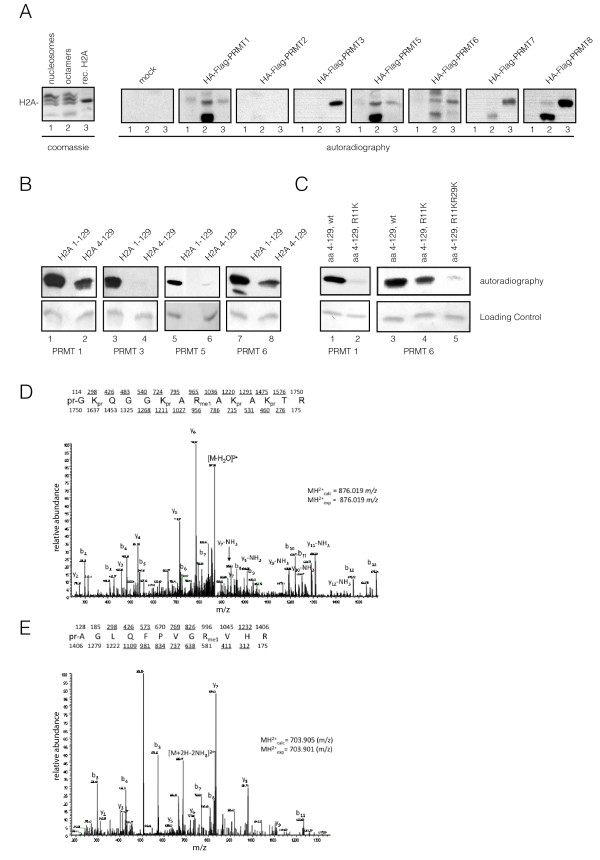
**Protein arginine methyltransferase (PRMT)1, 5 and 6 can methylate recombinant and endogenous H2A**. **(A) **Histone methyltransferase (HMT) assays with different Flag-hemagglutinin (HA)-tagged PRMTs immunoprecipitated from HEK 293, stably expressing PRMTs on (lane 1) nucleosomes, (lane 2) purified histones and (lane 3) recombinant full-length H2A. (Left) Coomassie stain and (right) autoradiography are shown. **(B) **HMT assays using recombinant full-length H2A and H2A 4-129. Only PRMT1 and 6 were found to methylate H2A 4-129. **(C) **HMT assays (left panel) with Flag-Ha-PRMT1 on H2A 4-129, H2A 4-129 R11 mutated to K, and (right panel) with Flag-Ha-PRMT6 on H2A 4-129, H2A 4-129 R11K, H2AR11K R29K. Loading controls are shown below. **(D) **Mass spectrometry (MS) analysis of H2 Amethylated *in vitro *by PRMT1 shows methylation of R11. Tandem MS (MS/MS) spectra of a propionylated histone H2A peptide at a mass:charge ratio (m/z) of 876 from trypsin digest of propionylated H2A is shown. **(E) **MS analysis of H2A, *in vitro *methylated by PRMT6 shows methylation of R29. MS/MS spectrum of a doubly-charged peptide ion at m/z of 703.901 is shown.

Given the overlapping target specificities displayed by some PRMT family members, we next addressed whether other PRMTs can methylate H2A. For this, we established HEK293 cell lines stably expressing Flag-hemagglutinin (HA) fusions of PRMT1, 2, 3, 5, 6, 7 and 8, and used immunoprecipitated PRMTs in our HMT assays (Figure 2A). Because PRMT4 is the best-studied PRMT and has been shown to methylate H3 only, we omitted it from our studies. We checked that the tagged PRMT1, 2, 3, 5, 6, 7, 8 were expressed (see Additional file 2, Figure S2A) and displayed the expected pattern of cellular distribution (see Additional file 2, Figure S2B). In addition we also verified that the immunoprecipitated PRMTs were active in our assays as shown by their ability to modify MBP (see Additional file 2, Figure S2C). In accordance with previous reports [18], PRMT2 showed no activity (Figure 2A; see Additional file 2, Figure S2C) in our assays, whereas PRMT1, 3, 5, 6, 7 and 8 could methylate recombinant H2A (Figure 2A, lane 3), but only PRMT1, 5 and 6 could also methylate H2A in octamers (Figure 2A, lane 2). We excluded PRMT7 and 8 from further investigations, because they co-immunoprecipitated with endogenous PRMT1 (data not shown), making it difficult to distinguish between their activities and those of PRMT1.

Next, we wanted to determine whether PRMT1, 3, 5 and 6 can methylate sites other than H2AR3. Importantly, we found that only PRMT1 and 6 (not PRMT3 and 5) had significant activity on H2A 4-129 (Figure 2B). To investigate which arginines can be methylated by these enzymes, we used H2A 4-129 with point mutations in different arginines in our HMT assays. For PRMT1, only background activity was detectable on a R11K (H2A4-129 R11K) mutant, indicating that it preferentially methylates H2AR11 (Figure 2C, left panel). For PRMT6 we saw a reduction in the methylation activity on a R11K mutant, but not a complete loss of the signal (Figure 2C, right panel), whereas on a R11KR29K (H2A4-129 R11KR29K) double mutant, only background activity was detectable, showing that in our HMT assays PRMT6 can methylate both H2AR11 and H2AR29.

To confirm these results on full-length H2A, we performed MS analysis after *in vitro *methylation by PRMT1 and 6. We found that PRMT1 does indeed methylate H2AR11 (Figure 2D), and that PRMT6 methylates H2AR29 (Figure 2E).

### H2AR29me2 is enriched on genes repressed by PRMT6

**Figure 3:**
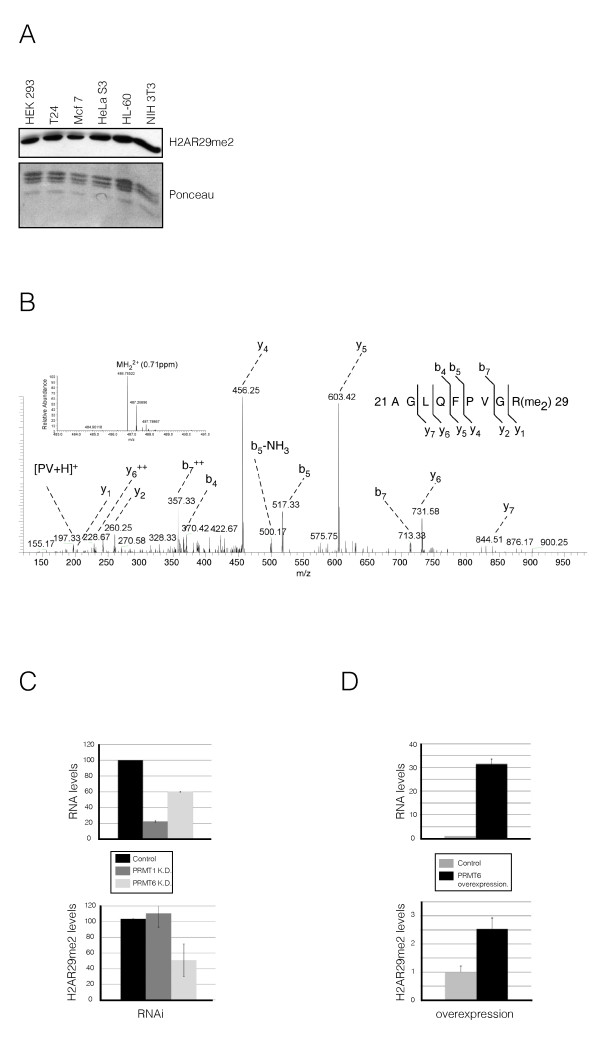
**H2AR29 is methylated by protein arginine methyltransferase (PRMT)6 *in vitro *and *in vivo***. **(A) **Immunoblot with H2AR29me2-specific antibody shows the presence of H2AR29me2 in various human and mouse cell lines. **(B) **Mass spectrometry (MS) analysis of endogenous, purified H2A. Shown in the collisionally activated dissociation (CAD) tandem MS (MS/MS) spectrum of the doubly-charged histone H2A derived tryptic peptide ^22^AGLQFPVGR(me_2_)^29 ^ion (mass:charge ratio (m/z) 486.78522; 0.71 ppm mass deviation), identifying R29 as dimethylated. The identity and assignment of the b and y ions of the dimethyl arginine-containing peptide was corroborated by nano liquid chromatography (LC)-MS/MS analysis of the corresponding chemically synthesised peptide (see Additional file 4, Figure S4). **(C) **(Top panel) H2AR29me2 levels decreased upon knockdown of PRMT6, but not PRMT1. PRMT1 RNA levels (relative to β-actin) measured by reverse transcriptase (RT)-PCR in HEK293 cells were reduced by approximately 80%, and PRMT6 by 50%. (Bottom panel) H2AR29me2 levels were not altered upon knockdown of PRMT1, but they decreased by approximately 50% upon PRMT6 knockdown. The average of the H2AR29me2 levels relative to the control from three independent experiments is shown; error bars correspond to the standard deviation of the means. **(D) **Overexpression of PRMT6 (top panel) in HEK293 cells resulted in increased H2AR29me2. (Top panel) PRMT6 RNA levels (relative to β-actin) were measured by RT-PCR. (Bottom panel) H2AR29me2 levels increased by approximately 2.5 times in PRMT6 overexpressing cells compared with the wild-type cells. The average of the H2AR29me2 levels relative to the control from four independent experiments is shown; error bars correspond to the standard deviation of the means.

To gain insight in the function of H2A methylation *in vivo*, we raised antibodies against asymmetrically dimethylated H2AR11 and H2AR29. We succeeded in generating a specific antibody that passed our rigorous quality testing for H2AR29me2 only (see Additional file 3, Figure S3A-D). Using this antibody, we detected H2AR29me2 in various human and mouse cell lines (Figure 3A). We also verified the presence of H2AR29me2 *in vivo *by MS analysis (Figure 3B; see Additional file 4, Figure S4A-C). To confirm that PRMT6 is indeed the enzyme responsible for H2AR29me2 *in vivo*, we used both a loss-of-function and a gain-of-function approach in HEK293 cells. Stable small hairpin RNA interference (RNAi)-mediated knockdown of PRMT6 (but not of PRMT1) resulted in a decrease in H2AR29me2 levels by approximately 50% (Figure 3C, left panel), whereas overexpression of PRMT6 increased H2AR29me2 levels by approximately 2.5 times (Figure 3C, right panel), clearly demonstrating the ability of PRMT6 to methylate H2AR29 *in vivo*.

**Figure 4:**
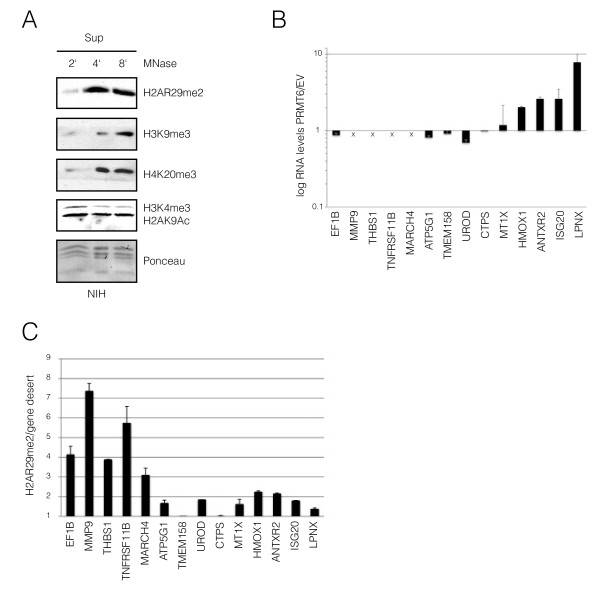
**H2AR29me2 is enriched at genes repressed by protein arginine methyltransferase (PRMT)6**. **(A) **H2AR29me2 was found to be enriched in less accessible chromatin fractions. Kinetic of micrococcal digestion of chromatin [28]. Kinetic of micrococcal digestion of chromatin [28] is shown. The abundance of the indicated histones modifications in the fractions was tested by western blotting with specific antibodies. **(B) **PRMT6 was overexpressed in HEK293 cells. Expression levels of the indicated genes were measured by real-time PCR and normalized to β-actin. The relative expression of the normalized genes in control and PRMT6-overexpressing cells was quantified using the ΔCt method, and the resulting relative quantification (RQ) values were plotted in a bar graph on a logarithmic scale. The cross indicates genes not expressed in HEK293 (GEO: record GSE11892; [20]). **(C) **Chromatin immunoprecipitation assay with H2AR29me2 antibody. Purified DNA from immunoprecipitated chromatin fragments from HEK293 cells was amplified by real-time PCR with specific primer sets for indicated gene promoters (see Additional file 6, Table S1). The enrichment of precipitated chromatin relative to an intergenic region was determined and plotted in a bar graph. Error (standard deviation) bars are shown.

To investigate the distribution H2AR29me2 in bulk chromatin, we performed a time course of micrococcal nuclease digestion of chromatin. Accessible chromatin enriched in active markers (H3K4me3, H2AK9Ac) is released in the early fractions, whereas condensed chromatin enriched in repressive markers (H3K9me3, H4K20me3) is only released at later time points. We detected H2AR29me2 enriched in the later fractions (Figure 4A), suggesting that it localises preferentially to transcriptionally inactive chromatin.

To determine whether H2AR29me2 has a function in PRMT6-dependent transcriptional regulation, we investigated whether H2AR29me2 is enriched at specific genes. Recently, by performing an expression profile after PRMT6 RNAi, PRMT6-regulated genes were identified [19], and we therefore concentrated on these genes. Of the genes tested, a subset (for example, HMOX1, ANTRX2, ISG20, LPNX) became upregulated upon PRMT6 overexpression (Figure 4B), suggesting an activating role of PRMT6 at these loci, whereas other genes (for example, EIF1B, ATP5G1, TMEM158, UROD) were (moderately) downregulated (Figure 4B). Several genes that did not react to PRMT6 overexpression have been described to be repressed by PRMT6 [19], but are actually transciptionally silent. Their mRNA was shown to be below the detection limit [20] (Figure 4B, cross).

Next we performed chromatin immunoprecipitation (ChIP) assays using our H2AR29me2-specific antibody. Interestingly, we found that H2AR29me2 was enriched on the promoters of genes that where found to be repressed by PRMT6 (for example, EIF1b, MMP9, THBS1, TNFRSF11B) (Figure 4C) [19], but we did not detect any significant enrichment at genes that are activated by PRMT6 (for example, HMOX1, ANTRX2, ISG20, LPNX) (Figure 4C). These data clearly implicate H2AR29me2 as a novel histone modification in PRMT6-mediated gene repression.

## Discussion

The results presented here show that PRMT1 and 6 can methylate histone H2A at arginine residues other than the previously described R3. We found that R11 and R29 in H2A can be methylated and are targeted for methylation by PRMT1 and 6, respectively. Furthermore, we found that *in vivo*, H2AR29me2 is enriched at genes that are repressed, but not at genes activated by PRMT6, implicating H2AR29me2 as a novel player in PRMT6-mediated gene repression.

### Systematic analysis of PRMTs for H2A specific activity

Currently, very little is known about the methylation status of H2A and its biological function. The first arginine in H2A, R3, has to date been the only methylation site characterised in H2A. H2AR3 (and H4R3) is within a GRG (glycine-arginine-glycine) motif, the consensus sequence for arginine methylation, explaining the observed strong activities towards H2AR3 in our *in vitro *assays. Recently, it has been shown that PRMT5 methylates cytoplasmic R3 of H2A rather than H4, and that it might be involved in the repression of differentiation genes [21]. However, because of the high sequence conservation between the extreme N-termini of H2A and H4, it is very difficult to distinguish between these two methylation sites *in vivo*.

We used a biochemical screen to identify H2A-specific HMTs and novel methylation sites. By applying different purification schemes, we found PRMT1 but no KMTs methylating H2A, in line with the fact that no H2A-specific KMT has been described to date. To investigate whether more members of the PRMT family can methylate H2A, we used PRMTs expressed in mammalian cells. We found that *in vitro*, PRMT1 and 6 can methylate the truncated form of H2A that lacks R3, showing that other sites are methylated. Through combined analysis by MS (Figure 3A, B), point mutants (Figure 3C) and Edman degradation (data not shown), we identified two new methylation sites in H2A: R11 and R29.

H2AR11 or H2AR29 are not within a GRG PRMT consensus sequence; however, arginines surrounded by small hydrophobic amino acids such as those for H2AR11 and R29, have also been shown to be good substrates for PRMTs [22]. To study the function of H2AR11me2 and H2AR29me2 *in vivo*, we attempted to raise specific antibodies. Although we were unable to obtain a satisfactory antibody for H2AR11me2, we managed to generate an antibody specific for H2AR29me2. Therefore we focused our *in vivo *studies on H2R29me2. Further studies will be required to determine the function of the second new methylation site, H2AR11me2.

### H2AR29 is a novel PRMT6 target

In *S. cerevisiae*, H2AR29 is conserved as H2AR30, however we have so far failed to detect H2AR30 methylation. In mammals, we found that PRMT6 can methylate H2AR29 *in vitro *and *in vivo*. PRMT6 is the primary enzyme responsible for H3R2 methylation [11,12], but has also been shown to methylate H4 and H2A at R3 [11]. H3R2 methylation has been well studied, and found to counteract the activating H3K4me3 marker, implicating it in the repression of genes [11,12,23]. Using an RNAi approach, genes regulated by PRMT6 were recently identified [19]. We analysed several of these genes for the presence of the novel H2AR29me2 marker. We found it to be enriched at the promoters of genes that were shown to be repressed by PRMT6, linking H2AR29me2 with transcriptional silencing by PRMT6, and giving novel insights into the mechanism of PRMT6-mediated gene repression. Of the H2AR29me2 target genes, thrombospondin-1 (*THBS1*) is of particular interest, being a potent natural inhibitor of angiogenesis and cell migration, dysregulated in several types of cancer [24]. Whereas DNA methylation has been shown to regulate *THBS1 *expression, the role of histone modifications is less understood [25], however, PRMT6 was recently shown to be present at the *THBS1 *promoter [19]. Our data suggest that the H2AR29me2 mark set by PRMT6 contributes to the regulation of *THBS1 *expression. Due to the lack of ChIP-grade PRMT6 antibodies, we cannot exclude that these genes are indirect PRMT6 targets; however, our PRMT6 knockdown and overexpression data strongly suggest that PRMT6 is the enzyme methylating H2AR29me2, and puts the marker at these genes.

In contrast to this repressive function, PRMT6 also has a function as a coactivator [26]. In line with this, we found genes that are activated by PRMT6 overexpression. Interestingly, we did not detect enrichment of H2AR29me2 on these genes. We therefore suggest that H2AR29me2 could help to discern the functional output of PRMT6 activity. Indeed, the ability of PRMT6 to act as a coactivator might be linked to its activity towards H4R3 and/or H2AR3 [8], rather than H2AR29. Our ChIP data, together with the preferential enrichment of H2AR29me2 in more inaccessible chromatin (Figure 4A), establish this novel marker as a potential key regulator in PRMT6-mediated gene repression.

## Conclusions

Our work has identified two new methylation sites in H2A and the corresponding methyltransferases. Importantly, we have revealed novel insights into the function of H2A methylation *in vivo *and in the mechanisms of PRMT6-mediated transcriptional repression. For the future it will be exciting to study further H2A modifications; however, this will require the development of novel specific antibodies. To unravel the multiple functions of PRMT6 [26], it will be important to investigate the mechanisms fine-tuning the activity of PRMT6 towards its targets on H3, H4 and H2A, and to identify the specific biological pathways involved in the deposition of H2A methylation markers.

## Methods

### Cloning of PRMT, H2A constructs and cell lines

PRMTs were cloned in pcDNA3.1 containing one Flag and two HA tags. Transfected HEK293 cells were selected in Dulbecco modified Eagle's medium (DMEM) containing G418 (PAA Laboratories GmbH, Pasching, Austria). Recombinant human H2A full-length and 4-129 constructs were cloned in pET21a.

### Antibodies and peptides

The following antibodies were used: H3K4me3 (Abcam Inc., Cambridge, MA, USA), H3K9me3 (Upstate Laboratories Inc., New York, NY, USA), H3K20me3 (Upstate), H2AK9Ac (Upstate), HA (12CA5) and Flag (Sigma Chemical Co., St Louis, MO, USA). The H2AR29me2 antibody was raised in rabbit against the peptide FPVGR(me2a)VHRLLGC. Additional peptides used were the unmethylated petide FPVGRVHRLLGC, the H2AR29 monomethylated peptide FPVGR(me1a)VHRLLGC, and the H2AR3 di-methylated peptide SGR(me2a)GKAGGC.

### Expression of recombinant H2A

Recombinant H2A constructs in pET21a were expressed in the Rosetta *Escherichia coli *strain, and induced with 0.4 mol/l isopropyl β-D-1-thiogalactopyranoside (IPTG) for 3 hours at 37°C. The preparation of inclusion bodies was performed as described previously [27]. H2A was dialysed against water for subsequent *in vitro *methylation assays.

### Extraction of endogenous histones from cells

Cells from different cell lines and PRMT knockdown cells were lysed in Ex-250 buffer (20 mmol/l HEPES pH 7.5, 250 mmol/l NaCl, 0.5 mmol/l MgCl_2_, 0.5% NP40) for 15 minutes on ice. After centrifugation the pellet was resuspended in 2% SDS and sonicated.

### Protein purification

For the purification of H2A methylating enzymes, approximately 8 × 10^9 ^HeLa S3 cells were collected. The cell pellet was resuspended in hypotonic buffer (20 mmol/l HEPES pH 7.5, 20 mmol/l NaCl, 5 mmol/l MgCl_2_). and incubated for 15 minutes on ice. After homogenization, the cytoplasmic fraction was removed by centrifugation. The nuclei were then lysed in hypotonic buffer + 0.5% NP-40 detergent for 30 minutes, and the soluble nuclear fraction was isolated by centrifugation for 30 minutes. After pre-clearing, the extract was adjusted to 100 mmol/l NaCl, and loaded onto a Q-Sepharose column (Pharmacia, Uppsala). Elution was performed with a stepwise increase of NaCl concentration, from 200 mmol/l to 1000 mmol/l NaCl. The H2A activity-containing fraction was diluted to 50 mmol/l NaCl, and loaded onto a diethylaminoethyl cellulose (DEAE)-Sepharose column (Pharmacia, Uppsala). The active fractions from the DEAE column were loaded onto a heparin-Sepharose column (Pharmacia, Uppsala), and factions from this column were dialysed against 20 mmol/l Tris pH 9 and 20 mmol/l NaCl. An *in vitro *methylation assay was then performed. The 400 and 600 mmol/l fractions from the heparin-Sepharose column were analysed by MS.

### MS analysis of heparin-sepharose fractions

For details see Additional file 5.

### Immunofluorescence

Stable cell lines expressing the different Flag-HA-PRMTs were seeded onto poly-L-lysine-treated coverslips, fixed in 4% paraformaldehyde/1% sucrose for 15 minutes, and permeabilised in PBS + 0.6% Triton for 5 minutes. Incubation with rabbit Flag antibody (Sigma, 1:200) and secondary antibody (Fluor 488 anti-rabbit IgG, Jackson Immunoresearch Laboratories, Inc., West Grove, PA, USA) was performed in PBS/0.1% Tween/3% BSA, then the cells were stained with 4',6-diamidino-2-phenylindole (DAPI) and mounted using mounting medium (Vectashield; Vector Laboratories Inc., Burlingame, CA, USA). Image acquisition was performed with a UV confocal microscope (SP2; Leica Microsystems Wetzlar GmbH, Wetzlar, Germany).

### Immunoprecipitation of Flag-HA-PRMTs

Stable cell lines, expressing individual PRMTs, were grown to confluency and lysed in Ex-250 buffer (20 mmol/l HEPES, pH 7.5, 250 mmol/l NaCl, 0.5 mmol/l MgCl_2_, 0.5% NP40), followed by centrifugation and dilution to 150 mmol/l NaCl. After another centrifugation step, HA antibodies (3 μg per 2 × 10^7 ^cells) were added, and incubated with 15 μl protein G sepharose. The beads were washed twice with Ex-150 buffer (20 mmol/l HEPES pH 7.5, 150 mmol/l NaCl, 0.5 mmol/l MgCl_2_, 0.5% NP40), and twice with PBS.

### *In vitro *methylation assays

An aliquot (2 μg) of core histones, recombinant H2A, MBP (Sigma) or glutathione S-transferase (GST)-GAR were incubated with 10 μl of immunoprecipitated PRMTs in PBS in a final volume of 30 μl. To this were added 0.5 μCu S-adenoslyl methionine (Amersham Laboratories, Amersham, Buckinghamshire, UK), then the mixture was incubated for 1 hour at 30°C, and reactions stopped by addition of loading buffer. Samples were loaded onto a 18.7% polyacrylamide gel, blotted onto a nitrocellulose membrane, and subjected to autoradiography.

### Reverse transcription and real-time PCR

Total RNA was extracted from cells (Trizol reagent; Invitrogen). cDNA was synthesised by using the MMLV reverse transcriptase (Fermentas) and oligodT primer according to the manufacturer's instructions. One-tenth of the cDNA was amplified by real-time PCR with SYBR Green detection using a thermocycler with fluorescence detection (7300; Applied Biosystems, Foster City, CA, USA) and primers (see Additional file 6, table S1).

### Western-blot quantification

ImageJ software was used for quantification of both Ponceau-stained bands and western-blot signals. The values corresponding to the western-blot signals were normalized to the total amount of histones loaded, and the ratios between the normalized signals in wild-type and PRMT1/PRMT6 knockdown cells or PRMT6 overexpressing cells, respectively, were calculated.

### ChIP

Cells were crosslinked with 1% formaldehyde for 2 minutes at room temperature, and the reaction was stopped by the addition of glycine to a final concentration of 0.25 mol/l. The fixed cells were rinsed twice with PBS, and resuspended in 1 ml of Lysis Buffer 1 (50 mmol/l Tris pH 8, 2 mmol/l EDTA pH 8, 0.1% NP40, 10% glycerol) per 4 × 10^7 ^cells. After 10 minutes on ice the lysate was centrifuged for 5 minutes at 500 *g*. The nuclear pellet was resuspended in 1 ml of Lysis Buffer 2 (50 mmol/l Tris pH 8, 10 mmol/l EDTA pH 8, 0.1% NP40, 0.2% SDS) and sonicated in a water-bath sonicator (Diagenode, Liege), then centrifuged at 21000 g for 10 minutes. The cleared supernatant was pre-cleared with 150 ml blocked beads for 1 hour, rotating at 4°C. Chromatin was diluted 10 times in ChIP Dilution Buffer (50 mmol/l Tris pH 8, 150 mmol/l NaCl, 0.25% NP40), and then incubated overnight at 4°C with 4 mg of the relevant antibody. The solution was then incubated for 2 hours with rotation at 4°C with 40 μl blocked beads to recover the bound material. The beads were washed, twice in 10 ml of Low Salt Buffer (20 mmol/l Tris pH 8, 150 mmol/l NaCl, 2 mmol/l EDTA pH 8, 0.25% NP40, 0.02% SDS), once with 10 ml of High Salt Buffer (20 mmol/l Tris pH 8, 250 mmol/l NaCl, 2 mmol/l EDTA pH 8, 0.25% NP40, 0.02% SDS), and eluted by two 15-minute incubations at 30°C with 125 μl elution buffer (1% SDS, 0.1 mol/l NaHCO_3_). Chromatin was reverse-crosslinked for 4 hours at 65°C in the presence of 10 U RNase (Roche Applied Science, Indianapolis, IN, USA) and 10 mg/ml proteinase K. DNA was extracted by phenol-chloroform, and further purified with a commercial kit (PCR Purification Kit; Qiagen Inc., Valencia, CA, USA) according to the manufacturer's instructions.

### MS/MS analysis of methylated H2A

For details see Additional file 5, additional methods

## Abbreviations list

BSA: bovine serum albumin; PBS: phosphate-buffered saline.

## Competing interests

The authors declare that they have no competing interests.

## Authors' contributions

TW and AI carried out most of the experimental work with the help of KK and CV. BMZ, BS, FR, BAG, HL and GM performed the mass spectrometry analysis. TW, AI, GM and RS participated in the design of the study. AI, TW and RS prepared the manuscript. All authors read and approved the final manuscript.

## Supplementary Material

Additional file 1 **Figure S1 - Identification of protein arginine methyltransferase (PRMT)1 as a histone H2A methyltransferase, using nanoscale liquid chromatography and mass spectrometry (MS)**. The two fractions (numbers 4 and 6) from the heparin column (Figure 1B) containing methyltransferase activity towards histone H2A 4-129 were analysed by MS as described (see Additional file 6, supplementary methods). Only proteins with two unique peptides possessing a total Mascot score of >54 were considered to be significant, and these were used for MS3 scoring in MSQuant software (http://msquant.sourceforge.net). PRMT1 was found in both heparin fractions. IPI and Uniprot accession numbers for PRMT1 are listed. Click here for file

Additional file 2 **Figure S2 - Purification and immunoprecipitation of the different protein arginine methyltransferases (PRMTs)**. **(A) **Immunoprecipitation of Flag-hemagglutinin (HA)-tagged PRMTs from stable cell lines with HA-specific antibodies. Western blots with HA antibodies are shown. Control for Figure 2A **(B) **Immunofluorescence with Flag-specific antibody of stable cell lines expressing Flag-HA PRMTs showing the localisation of the PRMTs. A merged view with 4',6-diamidino-2-phenylindole (DAPI) in blue and Flag signal in green is shown on the right. **(C) **Histone methyltransferase assay of the immunoprecipitated Flag-HA PRMTs using 2 μg myelin basic protein (MBP) shows that HA-Flag PRMTs 1, 3, 5, 6, 7 and 8 are active. (Upper panel) Autoradiography; (bottom panel) Coomassie stain (shown as loading control). Click here for file

Additional file 3 **Figure S3 - Characterisation of the novel H2AR29me2a antibody**. **(A) **The indicated amounts of unmodified H2AR29 peptide, monomethylated H2AR29 peptide, asymmetrically dimethylated H2AR29 peptide (aa 25 to 34 of H2A) and asymmetrically dimethylated H2AR3 peptide (aa 1 to 8) were spotted onto a membrane and probed with the H2AR29me2a antibodies. This antibody specifically recognised the immunising peptide in peptide dot blots, but not the unmodified or the H2AR3 dimethylated peptide. **(B) **Full-length recombinant H2A and equal amounts of acid-extracted histones (see Coomassie staining below) from HeLa and Mcf7 cells were loaded onto an SDS-PAGE gel and blotted with the H2AR29me2-specific antibody. (Upper panel) In the western blot, the H2AR29me2 antibody specifically recognised endogenous histones. **(C) **SDS-extracted histones were loaded onto an SDS-PAGE gel. H2AR29me2 antibody was incubated with no competitor, with 1 pmol unmethylated peptide, or with H2AR29me2 peptide. The methylated, but not the unmethylated peptide, competed for the signal, confirming the specificity of the antibody. In addition, the H3R17me2 peptide was not able to compete, showing that this antibody is specific for H2AR29me2. **(D) **Total nuclear extract (NIH-3T3 cells) was loaded onto an SDS-PAGE gel and (left panel) probed with H2AR29me2-specific antibodies; (right panel) Ponceau stain. In this whole-cell extract, the H2AR29me2 antibody specifically recognised H2A. Click here for file

Additional file 4 **Figure S4 - Identification of H2AR29me2 *in vivo *by mass spectrometry (MS) analysis**. **(A) **Overlay of the collisionally activated dissociation (CAD) tandem MS (MS/MS) spectra (derived from the endogenous H2A, isolated from Raji cells) of the specific tryptic (18 hour digest) peptide 22AGLQFPVGR(me2)29 (black) and the corresponding synthetic peptide (supplementary methods) (red). Both spectra are virtually identical, and allow the assignment of b and y ions and the internal fragment ion PV+H+. **(B) **Trypsin cleavage carboxyterminal to the dimethylated R29 in the histone H2A N-terminal tail is inefficient, and requires extended incubation times. Acid-extracted and gel-separated endogenous H2A was digested with trypsin for the time points indicated. and subsequently analysed by nano liquid chromatography (LC)-MS/MS. An extracted ion chromatogram (XIC; illustrated in red) of the peptide ion 22AGLQFPVGR(me2)29 (mass:charge ratio (m/z) 486.77), which eluted between 40.5 to 40.9 minutes from the C18 reverse-phase column, was derived from the total ion chromatogram (TIC; illustrated in black). **(C) **The peak area under the curve for the XIC of m/z 486.77 (mass was calculated and plotted against the trypsin digestion time points as indicated. An increase of more than 10-fold in peptide ion intensity was seen in the 18 hour compared with the 1 hour trypsin digest. Click here for file

Additional file 5 **Supplementary materials and methods**. Click here for file

Additional file 6 **Table S1: Primers used in this study**. Click here for file
