# Supplementary material for: Methylation of H2AR29 is a novel repressive PRMT6 target
Source: Epigenetics Chromatin. 2011 Jul 20;4:11. doi: 10.1186/1756-8935-4-11 (PMC3164600; doi:10.1186/1756-8935-4-11)
Supplement: Additional file 1 — Figure S1 - Identification of protein arginine methyltransferase (PRMT)1 as a histone H2A methyltransferase, using nanoscale liquid chromatography and mass spectrometry (MS). The two fractions (numbers 4 and 6) from the heparin column (Figure 1B) containing methyltransferase activity towards histone H2A 4-129 were analysed by MS as described (see Additional file 6, supplementary methods). Only proteins with two unique peptides possessing a total Mascot score of >54 were considered to be significant, and these were used for MS3 scoring in MSQuant software (http://msquant.sourceforge.net). PRMT1 was found in both heparin fractions. IPI and Uniprot accession numbers for PRMT1 are listed. [file 1756-8935-4-11-S1.PDF]

Additional Figure 1

Heparin Fraction 0.4 M NaCl

Total number of identified proteins: 142

Identified protein methyltransferase: PRMT1 (Protein arginine N-methyltransferase 1)

IPI Accession Number	Uniprot Accession Number	Peptides	Peptide Score
IPI00018522	Q99873-1	ATLYVTAIEDR	97
		QTVFYMEDYLTVK	99
		VEDLTFTSPFCLQVK	81
		VVLDVGSGTGILCMFAAK	113
		WLAPDGLIFPDR	97

Heparin Fraction 0.6 M NaCl

Total number of identified proteins: 128

Identified protein methyltransferase: PRMT1 (Protein arginine N-methyltransferase 1)

IPI Accession Number	Uniprot Accession Number	Peptides	Peptide Score
IPI00018522	Q99873-1	ATLYVTAIEDR	84
		EVDIYTVK	51
		LDHVVTIIK	82
		VIGIECSSISDYAVK	110
		VVLDVGSGTGILCMFAAK	96
		WLAPDGLIFPDR	125
