# Supplementary figures and images for: Methylation of H2AR29 is a novel repressive PRMT6 target
Source: Epigenetics Chromatin. 2011 Jul 20;4:11. doi: 10.1186/1756-8935-4-11 (PMC3164600; doi:10.1186/1756-8935-4-11)

Additional Figure 2

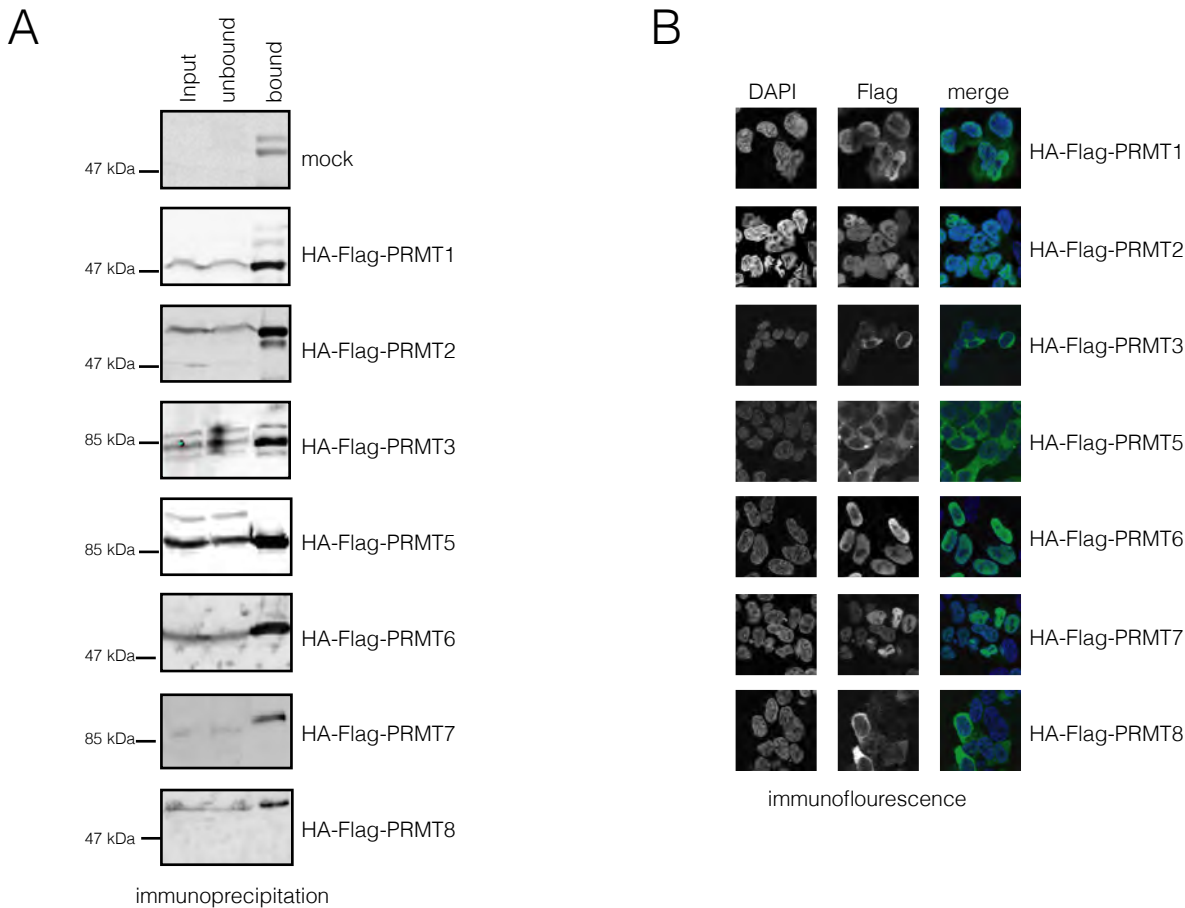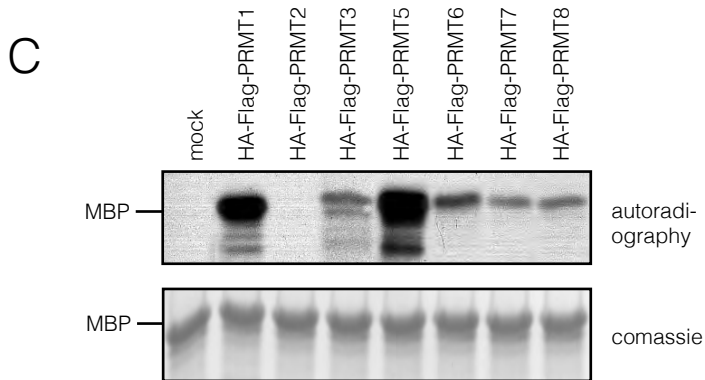

Supplement: Additional file 2 — Figure S2 - Purification and immunoprecipitation of the different protein arginine methyltransferases (PRMTs). (A) Immunoprecipitation of Flag-hemagglutinin (HA)-tagged PRMTs from stable cell lines with HA-specific antibodies. Western blots with HA antibodies are shown. Control for Figure 2A (B) Immunofluorescence with Flag-specific antibody of stable cell lines expressing Flag-HA PRMTs showing the localisation of the PRMTs. A merged view with 4',6-diamidino-2-phenylindole (DAPI) in blue and Flag signal in green is shown on the right. (C) Histone methyltransferase assay of the immunoprecipitated Flag-HA PRMTs using 2 μg myelin basic protein (MBP) shows that HA-Flag PRMTs 1, 3, 5, 6, 7 and 8 are active. (Upper panel) Autoradiography; (bottom panel) Coomassie stain (shown as loading control). [file 1756-8935-4-11-S2.PDF]

Additional Figure 3

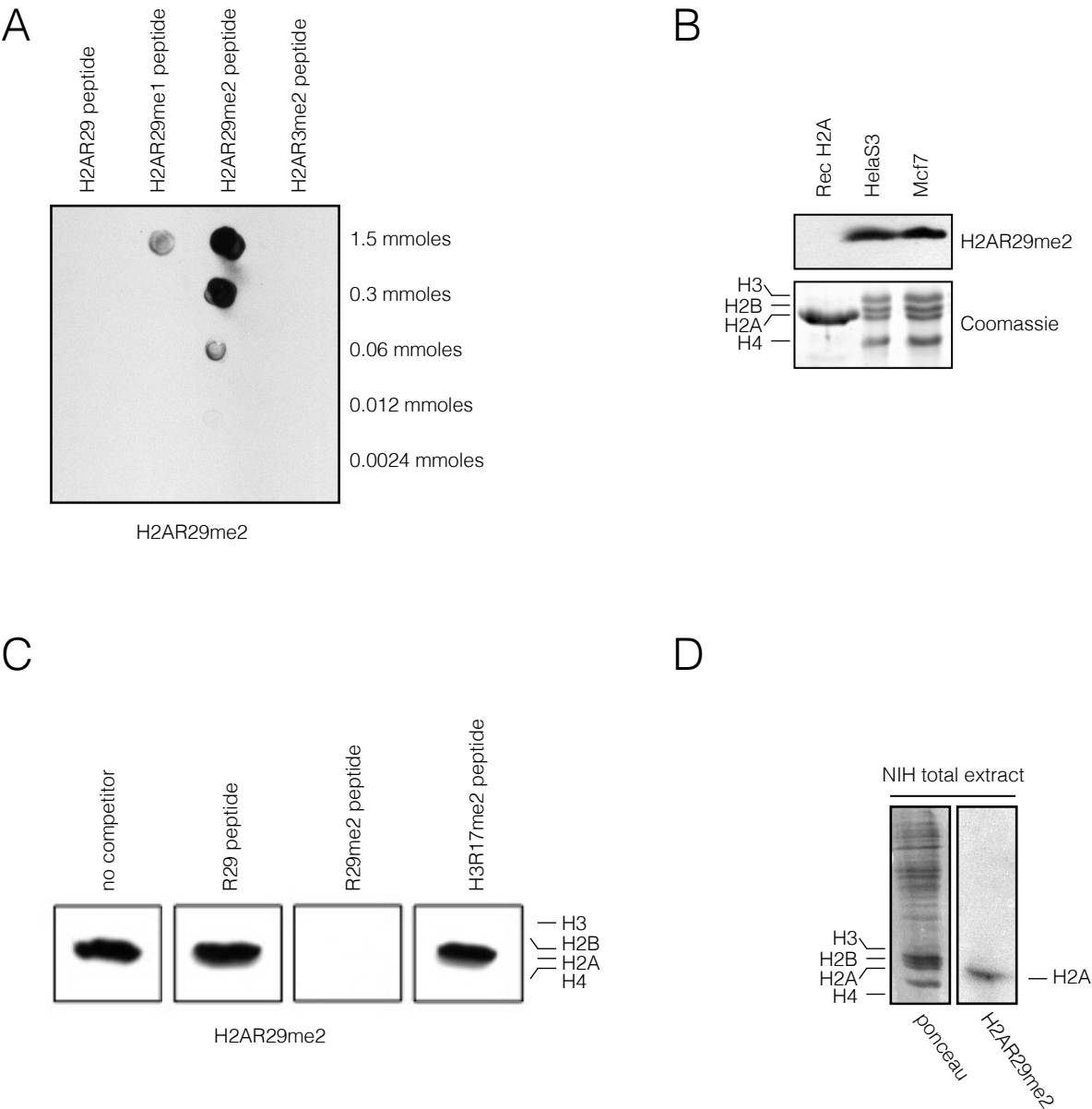

Supplement: Additional file 3 — Figure S3 - Characterisation of the novel H2AR29me2a antibody. (A) The indicated amounts of unmodified H2AR29 peptide, monomethylated H2AR29 peptide, asymmetrically dimethylated H2AR29 peptide (aa 25 to 34 of H2A) and asymmetrically dimethylated H2AR3 peptide (aa 1 to 8) were spotted onto a membrane and probed with the H2AR29me2a antibodies. This antibody specifically recognised the immunising peptide in peptide dot blots, but not the unmodified or the H2AR3 dimethylated peptide. (B) Full-length recombinant H2A and equal amounts of acid-extracted histones (see Coomassie staining below) from HeLa and Mcf7 cells were loaded onto an SDS-PAGE gel and blotted with the H2AR29me2-specific antibody. (Upper panel) In the western blot, the H2AR29me2 antibody specifically recognised endogenous histones. (C) SDS-extracted histones were loaded onto an SDS-PAGE gel. H2AR29me2 antibody was incubated with no competitor, with 1 pmol unmethylated peptide, or with H2AR29me2 peptide. The methylated, but not the unmethylated peptide, competed for the signal, confirming the specificity of the antibody. In addition, the H3R17me2 peptide was not able to compete, showing that this antibody is specific for H2AR29me2. (D) Total nuclear extract (NIH-3T3 cells) was loaded onto an SDS-PAGE gel and (left panel) probed with H2AR29me2-specific antibodies; (right panel) Ponceau stain. In this whole-cell extract, the H2AR29me2 antibody specifically recognised H2A. [file 1756-8935-4-11-S3.PDF]

A

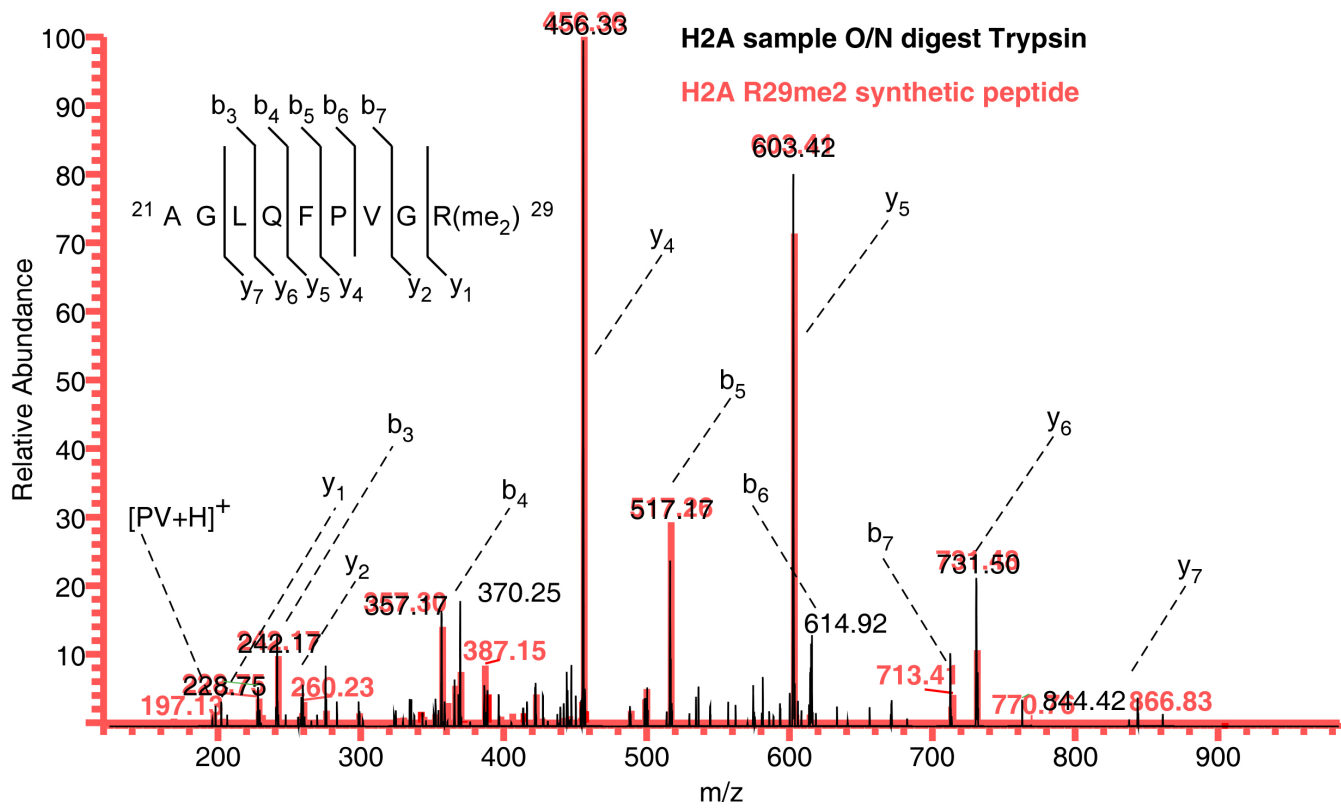

Additional Figure 4

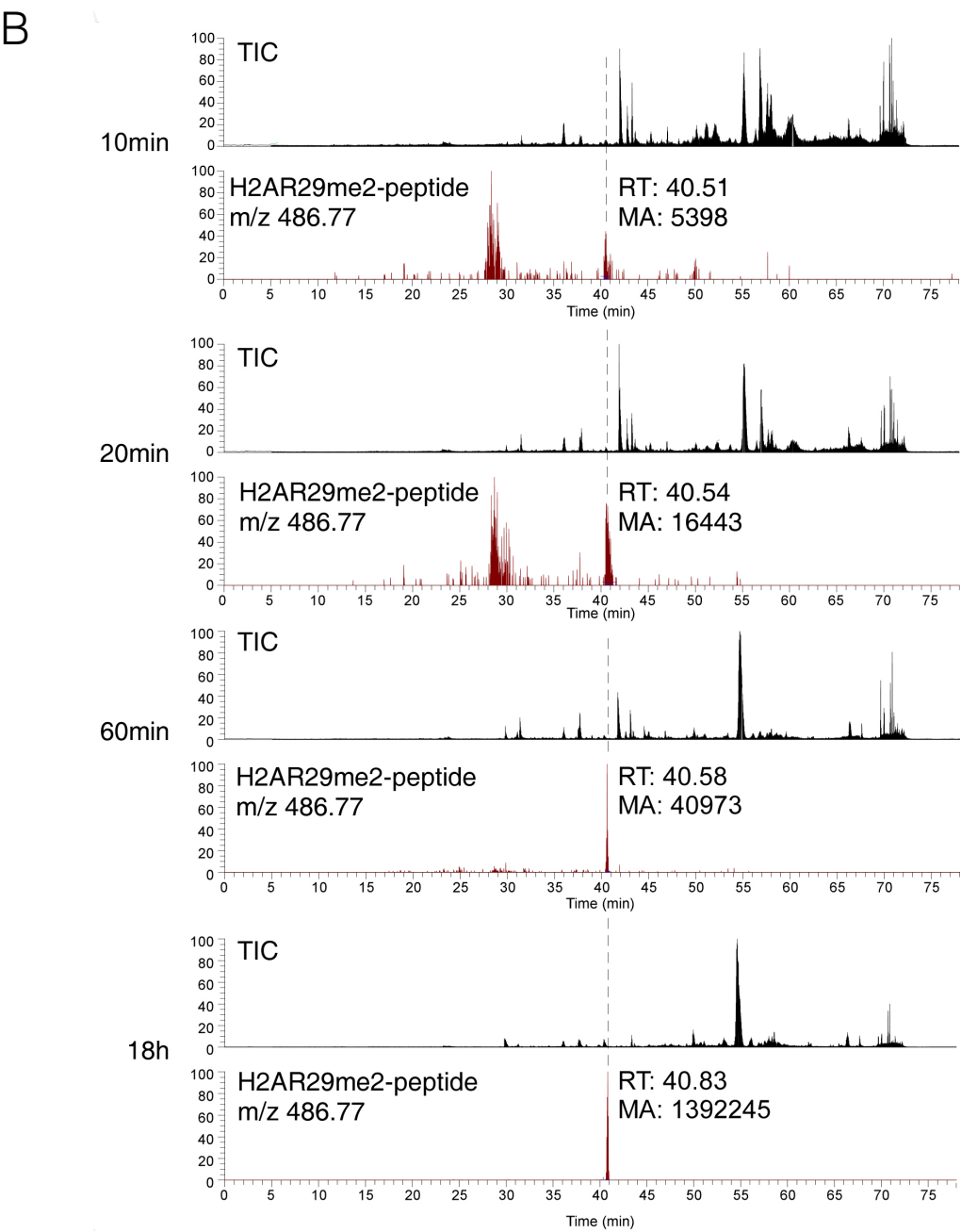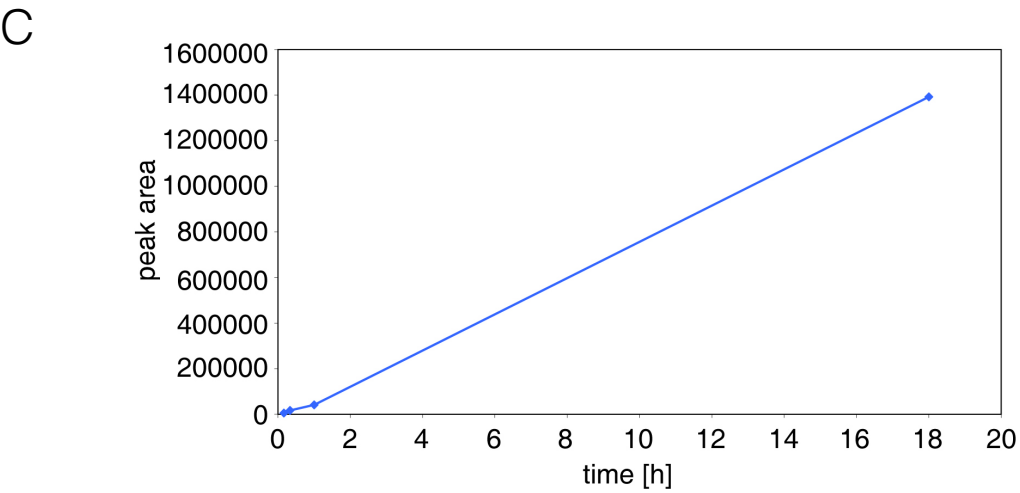

Supplement: Additional file 4 — Figure S4 - Identification of H2AR29me2 in vivo by mass spectrometry (MS) analysis. (A) Overlay of the collisionally activated dissociation (CAD) tandem MS (MS/MS) spectra (derived from the endogenous H2A, isolated from Raji cells) of the specific tryptic (18 hour digest) peptide 22AGLQFPVGR(me2)29 (black) and the corresponding synthetic peptide (supplementary methods) (red). Both spectra are virtually identical, and allow the assignment of b and y ions and the internal fragment ion PV+H+. (B) Trypsin cleavage carboxyterminal to the dimethylated R29 in the histone H2A N-terminal tail is inefficient, and requires extended incubation times. Acid-extracted and gel-separated endogenous H2A was digested with trypsin for the time points indicated. and subsequently analysed by nano liquid chromatography (LC)-MS/MS. An extracted ion chromatogram (XIC; illustrated in red) of the peptide ion 22AGLQFPVGR(me2)29 (mass:charge ratio (m/z) 486.77), which eluted between 40.5 to 40.9 minutes from the C18 reverse-phase column, was derived from the total ion chromatogram (TIC; illustrated in black). (C) The peak area under the curve for the XIC of m/z 486.77 (mass was calculated and plotted against the trypsin digestion time points as indicated. An increase of more than 10-fold in peptide ion intensity was seen in the 18 hour compared with the 1 hour trypsin digest. [file 1756-8935-4-11-S4.PDF]
