# Supplementary material for: Methylation of H2AR29 is a novel repressive PRMT6 target
Source: Epigenetics Chromatin. 2011 Jul 20;4:11. doi: 10.1186/1756-8935-4-11 (PMC3164600; doi:10.1186/1756-8935-4-11)
Supplement: Additional file 5 — Supplementary materials and methods. [file 1756-8935-4-11-S5.DOCX]

**Additional methods**

***MS analysis of Heparin Sepharose fractions***

Proteins eluted from the Heparin column fractions were separated on 4-12 % Bis-Tris SDS gels (NuPAGE, Invitrogen) and stained with silver or colloidal coomassie. The gels were sliced into ten equally sized gel pieces and subjected to tryptic in-gel digestion, essentially as described {Shevchenko, 2006 #6}. Prior to LC-MS analysis tryptic peptide mixtures were desalted using STAGE tips as described previously {Rappsilber, 2003 #1} with the following modifications: To ensure recovery of highly hydrophilic peptides, flow-through fractions from the C18 STAGE tips were further applied to Carbon STAGE tips (made from Empore 3M, activated carbon discs) and the pooled elutions were subsequently analyzed by LC-MS.

Nanoscale reversed phase LC (GE MDLC nanoflow) was coupled to a 7-Tesla linear ion-trap Fourier-transform ion cyclotron resonance mass spectrometer (LTQ-FT, Thermofisher, Germany) equipped with a nanoelectrospray source (Proxeon, Denmark). The 15 cm fused silica emitter (New Objective, USA) with an inner and spray tip diameter of 75 and 8 µm, respectively, was packed with reverse-phase ReproSil-Pur C18-AQ 3 µm resin (Dr. Maisch GmbH, Germany). Peptides were eluted by a linear gradient running from 2 to 42% (v/v) acetonitrile at a flow rate of 250 nl/min and sprayed directly into the orifice of the mass spectrometer. Information dependent acquisition of MS, MS/MS and MS^3^ spectra was performed essentially as described with minor modifications {Olsen, 2004 #12}. The general mass spectrometric conditions were: spray voltage, 2.3 kV; no sheath and auxiliary gas flow; ion transfer tube temperature, 120°C; collision gas pressure, 1.3 mTorr; normalized collision energy using wide-band activation mode; 30% for MS^2^ and 28% for MS^3^. Ion selection thresholds were 500 counts for MS^2^ and 5 counts for MS^3^. An activation q = 0.25 and activation time of 30 ms was applied in both MS^2^ and MS^3^ acquisitions.

MS data acquired on the LTQFT instrument were searched with Mascot 2.1 against the human International Protein Index protein database (IPI, version 3.12), to which we added frequently observed contaminants as described {Ong, 2004 #11}. The maximum allowed mass deviation MMD for monoisotopic precursor ions and MS/MS peaks were restricted to 5 ppm and 0.5 Da, respectively. Enzyme specificity was set to trypsin (with a maximum of 2 missed cleavages) allowing cleavage N‑terminal to proline and C‑terminal to aspartate. Protein identifications were manually verified by the use of MSQuant {Mortensen, 2010 #42}. For the interpretation of MS^3^ spectra, peak lists derived from Xcalibur 1.4 (Thermofisher) raw files were generated by DTA Super Charge software v.1.8, searched with Mascot 2.1 against the human IPI database and analyzed by the MS^3^ scoring function of MSQuant.

***MS/MS analysis of methylated H2A***

*In vitro* or *in vivo* modified histone H2A was propionylated, digested with trypsin and analyzed by nanoLC‑MS as previously published {Garcia, 2007 #5}. In addition we performed limited tryptic in-gel proteolysis experiments with H2A-containing gel bands (incubation times 5min, 10min, 15min, 20min, 30min, 60min and over night prior to sample clean-up). For the latter, sample desalting was achieved essentially as described before, except that the STAGE tip elution buffer B contained 50% instead of 80% acetonitrile. Desalted samples were subsequently analyzed using nanoflow (Agilent 1200 nanoLC, Germany) LC-MS/MS essentially as described above but on a linear ion trap-Orbitrap XL mass spectrometer (ThermoFisher, Germany). Peptides were eluted with a linear gradient of 2−60% buffer B (80% ACN and 0.5% acetic acid) at a flow rate of 250 nL/min over 40 or 60 min depending on the experiment. Data were acquired using a data-dependent “top 5” method, dynamically choosing the five most abundant precursor ions from the survey scan (mass range 350−1600 Th) in order to isolate and fragment them in the LTQ. Dynamic exclusion was defined by a list size of 500 features and exclusion duration of 30 s with a MMD of 10 ppm. Early expiration was disabled to decrease the resequencing of isotope clusters. The isolation window for the precursor ion selection was set to 2.0 Th. For the survey scan a target value of 1

000

000 (1000 ms maximal injection time) and a resolution of 60

000 at *m*/*z* 400 were set, whereas the target value for the fragment ion spectra was set to 10

000 ions (250 ms maximal injection time). The general mass spectrometric conditions were: spray voltage, 2.3 kV; no sheath and auxiliary gas flow; ion transfer tube temperature, 150°C; collision gas pressure, 1.3 mTorr; normalized collision energy using wide-band activation mode; 30% for MS^2^. Ion selection thresholds were 500 or 1000 counts for MS^2^ depending on the experiment. An activation q = 0.25 and activation time of 30 ms was applied.

MS data were processed into peak lists by DTASuperCharge 2.0b (part of the MSQuant 2.0b7 software environment) and searched with Mascot 2.2 against the human International Protein Index protein database (IPI, version 3.65) combined with frequently observed contaminants and concatenated with the reversed versions of all sequences.. The MMD for monoisotopic precursor ions and MS/MS peaks were restricted to 5 ppm and 0.5 Da, respectively. Enzyme specificity was set to trypsin (with a maximum of 3 missed cleavages) allowing cleavage N‑terminal to proline and C‑terminal to aspartate. Modifications were cysteine carbamidomethylation (fixed) as well as protein N-terminal acetylation, asparagine and glutamine deamidation and methionine oxidation (variable). For the detection of methylated lysine and arginine residues mono- and di-methyl arginine as well as mono-, di- and tri-methyl lysine were chosen as variable modifications. Protein and peptide identifications were further analyzed and manually verified by inspection of chromatograms and spectra.

In order to verify the unexpected trypsin cleavage C‑terminal to the di-methylated R29 in histone H2A the peptide H_2_N‑AGLQFPVGR(me_2_)‑CO_2_H was synthesized by standard Fmoc chemistry employing asymmetric di-methyl arginine (Biosyntan, Berlin) and analyzed by nanoLC‑MS/MS. The generated fragment spectra were overlaid and thereby compared to the MS/MS spectra of the corresponding peptide derived from endogenous H2A. In addition, nanoLC‑MS/MS analysis of a tryptic digest of the synthetic peptide (Biosyntan, Berlin) harboring the sequence H_2_N‑FPVGR(me_2_)VHRLLGC‑CO_2_H demonstrated the generation of H_2_N‑FPVGR(me_2_)‑CO_2_H.

**Supplemental references**

Garcia BA, Mollah S, Ueberheide BM, Busby SA, Muratore TL, Shabanowitz J, Hunt DF (2007) Chemical derivatization of histones for facilitated analysis by mass spectrometry. *Nat Protoc* **2:** 933-938

Mortensen P et al (2010) MSQuant, an open source platform for mass spectrometry-based quantitative proteomics. *J Proteome Res* **9:** 393-403

Olsen JV, Mann M (2004) Improved peptide identification in proteomics by two consecutive stages of mass spectrometric fragmentation. *Proc Natl Acad Sci U S A* **101:** 13417-13422

Rappsilber J, Ishihama Y, Mann M (2003) Stop and go extraction tips for matrix-assisted laser desorption/ionization, nanoelectrospray, and LC/MS sample pretreatment in proteomics. *Anal Chem* **75:** 663-670

Shevchenko A, Tomas H, Havlis J, Olsen JV, Mann M (2006) In-gel digestion for mass spectrometric characterization of proteins and proteomes. *Nat Protoc* **1:** 2856-2860
