# Supplementary material for: Methylation of H2AR29 is a novel repressive PRMT6 target
Source: Epigenetics Chromatin. 2011 Jul 20;4:11. doi: 10.1186/1756-8935-4-11 (PMC3164600; doi:10.1186/1756-8935-4-11)
Supplement: Additional file 6 — Table S1: Primers used in this study. [file 1756-8935-4-11-S6.PDF]

Table 1
Sequences of the primers used in the ChiPs and RT-PCR experiments. Protein symbol and GeneBank accession number are also listed

Protein names (human)	Protein symbols	GeneBank accession number	ChIP primers	RT PCR primers
eukaryotic translation initiation factor 1B	EIF1B	NM_005875.2	fw ACTTAGGGAAAGATGCGGATG rv TTAACCGTAACTGTGCCTTGC	fw TCCACTATCCAGAACCTCCAA rv GGACATTTGTCACACAACAGC
matrix metallopeptidase 9 (gelatinase B, 92kDa gelatinase, 92kDa type IV collagenase)	MMP9	NM_004994.2	fw CATGAGAAAGGGCTTACACCA rv CCCTTTCATACAGTTCCCACA	fw CAGGTTCGACGTGAAGGCGCAGATG rv CATAGGTCACGTAGCCCACTTGGTC
thrombospondin 1	THBS1	NM_003246.2	fw TAACGAATGGCTCTCTTGGTG rv TGGCGAGAGGTGGATACTAGA	fw TGCCTGATGACAAGTTCCAAG rv CCAGAGTGGTCTTTCCGCTC
tumor necrosis factor receptor superfamily, member 11b (osteoprotegerin)	TNFRSF11B	NM_002546.3	fw GAGTGTGTCCCAGACCTTTGT rv GGCCCTGTAATTTGAGGTTTC	fw GCGCTCGTGTTTCTGGACA rv AGTATAGACACTCGTCACTGGTG
membrane-associated ring finger (C3HC4) 4	MARCH4	NM_020814.1	fw GCTGCAAGAACCTTGTTTCC rv GCCAGGTGTTGTCTCGTCTT	fw CCTGTCCCCCTCTTTTAAGACT rv CTGGGGCACACAATCCATAG
ATP synthase, H+ transporting, mitochondrial F0 complex, subunit C1 (subunit 9)	ATP5G1	NM_005175.2	fw CTCTATGGTCGAGCGTTTCAG rv TCCCCACTCTTATTGGCTTTC	fw TCCAGCTCTGATCCGCTGT rv GTGGGAAGTTGCTGTAGGAAG
transmembrane protein 158 (RIS1)	TMEM158	NM_015444.2	fw GGAGACCATCCTTTTGTCAGG rv GACCTCAGTTTCCCCTTCTGT	fw GCTGCCTAGACTTCAGCCTG rv CGCTCCACACCACGATGAC
uroporphyrinogen decarboxylase	UROD	NM_000374.3	fw TCCCCAGTCTTGTAGCTCCTT rv GTCTTATCCAGCCAGGTCCAT	fw ATGGAAGCGAATGGGTTGGG rv CGGGTTTCCCTAAACTCTGGTA
CTP synthase	CTPS	NM_001905.1	fw GTGTGCCTCCCTACATCGTTA rv CTAGGATCCCGAAAAGTACGG	fw CAGTGTGGGCACAATACTCAA rv ACAAAAACCTCACCATGCTCATA
metallothionein 1X	MTX	NM_005952.2	fw TAAGAGAAACGTGGCCAACAG rv AATGAGAGGCAGAGGATGTGA	fw GTTTTCCTCTTGATCGGGAAC rv CACTTGTCTGACGTCCCTTTG

heme oxygenase (decycling) 1	HMOX1	NM_002133.1	fw AGAGCCTGCAGCTTCTCAGAT rv ACCCTTGGGAAACAAAGTCTG	fw GTCTTCGCCCCTGTCTACTTC rv CTGGGCAATCTTTTTGAGCAC
Anthrax toxin receptor 2	ANTRX2	NM_058172.3	fw AGTCTTGGGTTTGTGAACCAC rv GGGAAAGGGAAGAAGGAGTTT	fw TTGCTCTGACAGATGGCAAGT rv GAGGGCTGCAATTCTAGGATTT
interferon stimulated exonuclease gene 20kDa	ISG20	NM_002201.4	fw CACTCCCACCACAAGTTTCTC rv CAGGGAATGTCCCACAACTAA	fw TCTACGACACGTCCACTGACA rv CTGTTCTGGATGCTCTTGTGC
leupaxin	LPNX	NM_004811.1	fw ACTTAACCAAGGGCATTCTGG rv ACTACCTTTCCCGTCGTGACT	fw CATGGCTCACCTGACTGAGAT rv GTTTCTGGCAGGATGCACAA
Intergenic region			fw rv	
b-actin	ACTB	NM_001101		fw CGGTTGGCCTTGGGGTTCAGGGGG rv ATCGTGGGGCGCCCCAGGCACCA
protein arginine methyltransferase 1	PRMT1	NM_198318.2		fw CTATGCCCGGGACAAGTGGC rv CAGCTGTTTGGGGTCCACGA
protein arginine methyltransferase 6	PRMT6	NM_018137.1		fw TCCCTGGAGGGGAGTCGGAG rv CCACTTTGTAGCGCAGCAGC
